# Cognition During and After Multiple Sclerosis Relapse as Assessed With the Brief International Cognitive Assessment for Multiple Sclerosis

**DOI:** 10.1038/s41598-018-26449-7

**Published:** 2018-05-25

**Authors:** Natasa Giedraitiene, Gintaras Kaubrys, Rasa Kizlaitiene

**Affiliations:** Clinic of Neurology and Neurosurgery, Institute of Clinical Medicine, Faculty of Medicine, Vilnius University, Vilnius, Lithuania

## Abstract

There is some evidence that cognition may be impaired during multiple sclerosis (MS) relapse. The aims of this study were to assess the cognitive status with the Brief International Cognitive Assessment for Multiple Sclerosis (BICAMS) in MS patients during relapse, in stable patients, and in healthy controls; to evaluate cognitive changes up to 3 months after relapse; and to estimate the impact of different factors on cognition after relapse. BICAMS was performed in 60 relapsing, 30 stable patients and 30 controls. Relapsing MS patients were assessed during relapse and one and three months after relapse. SDMT score was lower in relapsing than in stable patients. The mean scores of all BICAMS tests were higher one month after relapse than during relapse (p < 0.001). SDMT score after relapse improved in younger patients, who had more severe relapse (p < 0.05). BVMT-R score improved more in men, in patients with biologically active interferon-beta, in patients treated with methylprednisolone and in patients who were rehabilitated (p < 0.05). CVLT-II score improved in women and in patients with shorter relapse (p < 0.05). A neuropsychological assessment, like the evaluation of physical disability, is important during relapse. BICAMS may be suitable for a quick and effective assessment of cognition during relapse.

## Introduction

Cognitive impairment (CI) occurs in up to 70% of multiple sclerosis (MS) patients. Neurocognitive dysfunction involves all of the subtypes of MS and is even found in the early stages of MS and clinically isolated syndromes^1,2^. Cognitive disturbances in MS patients have a negative impact on the lives of MS patients and the lives of their families. CI is often the leading predictor of occupational disability in MS patients, even when the physical disability is quite low^3–5^. CI occurring in the early stages of the disease predicts disability progression and earlier conversion to secondary progressive MS (SPMS) in newly diagnosed relapsing remitting MS (RRMS) patients^6,7^.

Despite the high prevalence of CI in MS patients, the importance of CI and its negative influence on patient’s employment, disability and prognosis, the cognitive assessment and follow-up of cognitive status are not performed regularly. In recent decades, the assessment of cognitive decline related to MS has received increasing attention. Many different neuropsychological batteries have been proposed. The first and the most frequently used batteries were the Brief Repeatable Battery of Neuropsychological tests (BRB-N)^8^ and Minimal Assessment of Cognitive Function in MS (MACFIMS)^9^. While both batteries are highly specific for the evaluation of CI in MS patients, their implementation in everyday clinical practice remains limited due to their high time demand and the need for surveillance and an interpretation by professional neuropsychologists. Therefore, considerable efforts have been made over the past decade to create a simpler but specialized neuropsychological instrument for the assessment of CI in MS patients. The result was the Brief International Cognitive Assessment for Multiple Sclerosis (BICAMS) battery^10,11^. BICAMS is a short and highly sensitive assessment battery; it is easily administered, does not require any special equipment or training, and may be conveniently used in everyday clinical practice^10–12^. Despite its simplicity, BICAMS is not readily available in most countries, although it is widely recognized that the assessment and follow-up of cognitive status, as well as treatment (in case of deterioration), should be as much a priority as the evaluation and treatment of physical disability in MS patients^10,11^.

Studying cognition during MS relapses is another hotly debated issue. Usually, MS relapses are diagnosed by a neurological examination and quantified with the Expanded Disability Status Scale (EDSS)^13^. EDSS is quite sensitive for assessing the patient’s ability to move and remains the gold-standard measure for assessing the level of disability^13,14^. However, EDSS is insensitive to the patient’s cognitive abilities and does not predict an accumulated cognitive disability^13–15^. Cognition might also be affected, and so-called cognitive relapses are hypothesized to occur during MS relapse^16–19^. It seems that the main cause of the deterioration in cognition is not usually detected, possibly due to the lack of appropriate assessment tools and the absence of routine and baseline assessments. From the clinical and scientific points of view, it is useful to examine the neurocognitive status along and with other aspects of neurological disability during MS relapse and to enhance the understanding of the clinically meaningful changes in cognition outcomes that may occur as a result of neurological worsening or response to treatment.

The aims of the study were (a) to assess the cognitive status according to BICAMS of MS patients during disease relapse, of stable MS patients, and of healthy controls; (b) to detect significant cognitive changes up to 3-months after relapse; and (c) to estimate the impact of different demographic, clinical and immunological factors on the cognition after relapse.

## Results

A total of 120 subjects were included in the study: 90 patients with clinically definite MS (60 relapsing (MSr) and 30 stable (MSs)) and 30 healthy control subjects (CG). All three groups were matched in demographic characteristics (Table 1).

**Table 1:** Demographic characteristics of MS patients and controls.

	MSr patients	MSs patients	CG	Test
Number of subjects, N	60	30	30	—
Gender Men/Women, N	21/39	13/17	12/18	χ^2^ = 0.635 p = 0.728
Age (years) Mean ± SD (range)	38.43 ± 9.6 (18–61)	37.47 ± 10.3 (22–59)	37.63 ± 9.5 (24–56)	ANOVA F = 0.13 p = 0.883
Education (years) Mean ± SD	14.80 ± 2.3	14.88 ± 2.8	15.42 ± 1.8	ANOVA F = 0.877 p = 0.419

MSr – relapsing MS patients, MSs – stable MS patients, CG – control group.

The MSr and MSs groups were matched for disease characteristics: disease duration, level of disability (assessed 3 months before and 3 month after relapse/baseline), mean number of exacerbations, period of remission and duration of immunomodulatory treatment (Table 2).

**Table 2:** Clinical characteristics of MS patients.

	MSr patients	MSs patients	p*
Number of subjects, N	60	30	—
Duration of the disease (years) Mean ± SD	8.94 ± 7.2	8.30 ± 7.5	0.697
EDSS (in MSr during the 3^rd^ month before the relapse)	3.59 ± 1.3 (N = 49**)	3.25 ± 1.19	0.243
EDSS (in MSr during the 3^rd^ month after the relapse)	3.78 ± 1.26	3.25 ± 1.19	0.061
The mean number of exacerbations	4.82 ± 3.3	3.83 ± 2.4	0.108
The period of remission (months) CI [lower-upper limit]	23.22 ± 23.80 [17.07–29.37]	30.2 ± 29.18 [19.31–41.10]	0.228
The duration of immuno-modulatory therapy (years) CI [lower-upper limit]	3.40 ± 3.42 [2.49–4.31]	3.33 ± 3.41 [2.06–4.61]	0.931

MSr – relapsing MS patients, MSs – stable MS patients, EDSS – Expanded Disability Status Scale, CI – confidence interval.

*Student t-test.

**It was possible to assess the EDSS score retrospectively in 49 relapsing MS patients.

Twenty-nine (48.3%) MSr patients were treated with intravenous methylprednisolone, 6 (10.0%) with plasma exchange and 25 (41.7%) with methylprednisolone and plasma exchange. Forty three patients (71.6%) were treated with methylprednisolone over a period of 3 days, 6 patients (10.0%) over a period of 4 days, 4 patients (6.7%) over a period of 5 days and 1 patient (1.7%) over a period of 6 days. Thirty-seven (61.7%) patients received rehabilitation after the relapse treatment, and 23 (38.3%) did not.

Longer durations of relapse (the period from the first neurological symptoms until the relapse treatment) were significantly associated with older age (r = 0.296, p = 0.022) and lower education (r = −0.260, p = 0.045). The longer the duration of relapse, the higher the disability increase (r = 0.287, p = 0.045) and, after relapse treatment, the less the EDSS score change (r = −0.320, p = 0.013).

A total of 240 tests were performed with BICAMS: relapsing MS patients were tested three times (during relapse and one and three months after relapse) and stable MS patients and CG were tested once. The mean scores of all three BICAMS tests (SDMT, BVMT-R and CVLT-II) and CVLT-II-delayed recall were compared between MSr, MSs and CG. The scores were significantly lower in MS patients (relapsing and stable) than in CG. The most affected cognitive domain in relapsing MS patients was verbal learning: the mean CVLT-II score was 14.95 points lower in relapsing MS patients than in CG. Less affected were the information processing speed and visuospatial learning and memory: the mean scores in relapsing MS patients were, respectively, 13.65 and 6.46 points lower than those in CG. The mean SDMT score was lower by 6.42 points in relapsing MS patients than in stable MS patients. However, no significant difference was found in the mean score of BVMT-R, CVLT-II or CVLT-II delayed recall between relapsing and stable MS patients (Table 3).

**Table 3:** Mean scores of BICAMS tests in MS patients and controls.

Test	MSr group (N = 60)	MSs group (N = 30)	CG (N = 30)	ANOVA	Post-hoc
SDMT	40.18 ± 11.42	46.60 ± 11.54	53.83 ± 8.91	F = 16.07; p < 0.001	CG > MSs > MSr^*^
BVMT-R (0–36)	22.57 ± 6.07	25.23 ± 5.66	29.03 ± 4.22	F = 13.63; p < 0.001	CG > MSs,MSr MSs = MSr^*^
CVLT-II (0–80)	52.02 ± 9.61	54.80 ± 9.40	66.97 ± 4.68	F = 30.99; p < 0.001	CG > MSs,MSr MSs = MSr^**^
CVLT-II delayed recall (0–16)	12.23 ± 2.66	12.80 ± 3.12	15.40 ± 0.72	F = 16.86; p < 0.001	CG > MSs, MSr MSs = MSr^**^

BICAMS - Brief International Cognitive Assessment for MS, MSr – relapsing MS patients, MSs – stable MS patients, CG – control group, SDMT - Symbol Digit Modalities Test, BVMT-R – Brief Visuospatial Memory Test-Revised, CVLT-II – California verbal learning test, II ed.

ANOVA – one-way analysis of variance.

^*^Bonferroni test was used for post- hoc analysis.

^**^Tamhane test was used for post -hoc analysis.

After comparing the mean results of BICAMS in MS patients and CG, the mean scores during relapse and at one and three months after relapse were compared in relapsing MS patients. The mean scores of all three BICAMS tests were significantly higher one month after relapse than during relapse: the mean score of SDMT was higher by 6.02 ± 6.14 points, BVMT-R by 3.73 ± 4.93 points, CVLT-II by 6.43 ± 6.00 points and CVLT-II delayed recall by 1.48 ± 1.94 points one month after relapse than during relapse. The mean score of CVLT-II was significantly higher by 2.12 points three months than one month after relapse. There was no significant difference in the result of SDMT or BVMT-R assessed three vs. one month after relapse in relapsing MS patients (Table 4).

**Table 4:** Mean scores of BICAMS tests during and after MS relapse.

Test	MSr group during relapse (MSr1) (N = 60)	MSr group 1 month after relapse (MSr2) (N = 60)	MSr group 3 months after relapse (MSr3) (N = 60)	ANOVA	Post hoc
SDMT	40.18 ± 11.42	46.20 ± 12.28	46.62 ± 10.96	F = 43.08; p < 0.001^*^	MSr1 < MSr2,MSr3 MSr2 = MSr3
BVMT-R (0–36)	22.57 ± 6.07	26.30 ± 4.56	26.93 ± 4.62	F = 34.73; p < 0.001^*^	MSr1 < MSr2,MSr3 MSr2 = MSr3
CVLT-II (0–80)	52.02 ± 9.61	58.45 ± 8.36	60.57 ± 9.59	F = 65.87; p < 0.001	MSr1 < MSr2 < MSr3
CVLT-II delayed recall (0–16)	12.23 ± 2.66	13.72 ± 2.22	13.98 ± 2.04	F = 33.84; p < 0.001^*^	MSr1 < MSr2,MSr3 MSr2 = MSr3

MSr1 – relapsing MS patients during relapse, MSr2 – relapsing MS patients one month after relapse, MSr3 – relapsing MS patients three months after relapse, SDMT – Symbol Digit Modalities Test, BVMT-R – Brief Visuospatial Memory Test-Revised, CVLT-II – California verbal learning test, II ed.

^*^Repeated-measures analysis of variance (rmANOVA) with Greenhouse-Geisser correction for violation of sphericity was used.

To assess the impact of different demographic, clinical and immunological factors on cognition after relapse, the differences between the BICAMS test results from relapse to one or three months later were calculated. Thus, the new measures were SDMT(1-R), SDMT(3-R), BVMT-R(1-R), BVMT-R(3-R), CVLT-II(1-R) and CVLT-II(3-R). Linear regression was used to assess and explain the relationship between each new cognitive parameter and the different independent cognitive and non-cognitive factors (demographic, clinical and immunological): age, gender, education, working status, course of the disease, duration of the disease, EDSS score before the relapse, EDSS scores during and after relapse, deterioration of the EDSS score before relapse and improvement after relapse, duration of relapse, treatment for relapse (methylprednisolone/plasmapheresis/rehabilitation), time to the initiation of immunomodulatory treatment after relapse, duration of the immunomodulatory therapy, biological activity of interferon-beta, relapse rate, and duration of remission before relapse. Linear regression was used. Significant independent variables (p < 0.05) were included in the models (Table 5).

**Table 5:** Regression models predicting cognitive changes after MS relapse.

Dependent variable	Regression models	R^2^	P (R^2^; coefficients)
SDMT(3-R)	11.72 − 0.29 × SDMT-R + 0.61 × education − 0.12 × age	0.243	<0.001
SDMT(3-R)	7.06 − 2.98 × EDSS(S-R) − 0.12 × SDMT-R	0.311	<0.001
SDMT(3-R)	5.61 + 3.09 × EDSS-R − 2.79 × EDSS-S − 0.12 × SDMT-R	0.306	<0.001
SDMT(3-R)	18.27 − 4.12 × IMG-R − 0.16 × SDMT-R	0.153	=0.042
BVMT-R(1-R)	23.77− 0.52 × BVMT-R-R − 3.1 × gender − 2.2 × rehabilitation	0.563	<0.001
BVMT-R(3-R)	20.98 − 0.54 × BVMT-R-R − 3.98 × methylprednisolone	0.483	<0.001
BVMT-R(3-R)	24.87 − 0.67 × BVMT-R-R − 3.01 × biological activity of IFN-beta	0.755	<0.001
CVLT-II(1-R)	15.49 − 0.27 × CVLT-II-R + 1.99 × gender	0.286	<0.001
CVLT-II(3-R)	30.40 − 0.37 × CVLT-II -R − 0.46 × duration of the relapse	0.292	<0.001

R^2^- coefficient of determination, SDMT(3-R) – the difference in SDMT results between three months and relapse, SDMT-R – SDMT result during relapse, EDSS(S-R) – deterioration in EDSS score (from the remission up to the relapse), EDSS-R – EDSS score during relapse, EDSS-S – EDSS score during the remission (three months before relapse), IMG-R – initiation of immunomodulatory therapy during the first month after relapse, BVMT-R(1-R) – difference in the BVMT-R results between one month and relapse, BVMT-R-R - BVMT-R result during relapse, BVMT-R(3-R) – difference in the BVMT-R results between three months and relapse, CVLT-II(1-R) – difference in the CVLT-II results between one month and relapse, CVLT-II-R – the CVLT-II result during relapse, CVLT-II(3-R) – difference in the CVLT-II results between three months and relapse.

In all models, some of the most important variables were the results of the BICAMS test during relapse: SDMT-R, BVMT-R-R or CVLT-II-R. The difference in BVMT-R after relapse significantly improved in men, while the difference in CVLT-II significantly improved in women. Gender had no significant impact on the SDMT test results. However, the SDMT score improved in younger patients with a higher level of education. SDMT was the only test for which the results were impacted by the EDSS score during the relapse and remission periods or the mean EDSS deterioration (the difference in the EDSS score from remission and relapse). The treatment options and treatment-related indicators had an impact on the difference in the BVMT-R score after relapse: better improvement was found in patients treated with methylprednisolone, but not with plasma exchange; in patients who had biologically active interferon-beta, and in patients who were rehabilitated after relapse, while a higher difference of the CVLT-II was estimated in patients with shorter relapse (or earlier treatment of relapse).

## Discussion

Impaired cognition has a negative impact on the quality of life of MS patient. However, in clinical practice, cognitive impairment is often underestimated. A physical disability is obvious during disease relapse, and it seems that deterioration in cognitive status can also occur^16–18^. Most likely, the main difficulty in the assessment of cognition during relapse is the lack of suitable and sensitive assessment methods of cognition. Additionally, the absence of routine and baseline assessments in routine clinical practice complicate the further evaluation and detection of cognition deterioration during relapse. EDSS is a sensitive and suitable assessment tool of the physical status and physical disability in MS patients but is quite insensitive for assessing cognition. The BICAMS battery was suggested to be an optimal, reliable and sensitive assessment tool for performing a quick cognitive evaluation in MS patients^10,11^, and it has been used by many researchers^20–25^. We decided to assess cognition during MS relapse and to measure its changes after relapse with BICAMS. This study is the first to assess cognition with BICAMS not only in relapsing MS patients but also in stable MS patients and healthy controls. Relapses were established by EDSS, and the specific non-cognitive symptoms that could compromise the cognitive assessment, such as optic neuritis, severe paresis or ataxia, fatigue, depression and other comorbidities, were excluded. The validity and reliability of the Lithuanian version of the BICAMS battery have been shown in our previous article^23^.

The results of the current study reveal that the mean scores of all of the BICAMS tests in MS patients were similar to our previous results: the SDMT, BVMT-R and CVLT-II results were worse in MS patients, both relapsing and stable, than in healthy controls^23^. Only the score of SDMT, which reflects the information processing speed, was worse in relapsing MS patients than in stable MS patients. Our study confirms the results of earlier publication^16^. In the study of Benedict *et al*., which assessed the SDMT, BVMT-R and PASAT tests during relapse, only the SDMT score was significantly lower in relapsing MS patients than in stable MS patients and the SDMT score between two assessments, before relapse and during the 3^rd^ month after relapse, did not differ^16^. In our study, the results of visuospatial and verbal memory tests, as in their study^16^, did not significantly differ between relapsing and stable MS patients. Our and other data^26–28^ confirm that SDMT is useful in neuropsychological MS research. In contrast to other cognition tests, SDMT has excellent test–retest reliability, is more sensitive to MS cognitive disorder, and correlates well with MRI parameters, such as lesion burden and atrophy^26–28^. The SDMT findings suggest that of all of the BICAMS tests, SDMT may be the most suitable to identify MS patients who experience cognitive relapse.

Assessing the results after relapse, we found that the results of all of the tests were significantly higher at one month after relapse than during relapse and that the results of only the verbal memory test were significantly higher at three months after relapse than after one month. It seems that improvements in information processing speed, visuospatial memory, and verbal memory can occur in MS patients during the first month after relapse. Nevertheless, an improvement of verbal memory was observed up to three months after relapse. Of course, it cannot be denied that an improvement of the CVLT-II score at three months was found due to the learning effect, as mentioned before; the same, recommended worldwide version of the test was used during relapse and three months after the treatment.

Summing the BICAMS results in relapsing MS patients, the more impaired scores of SDMT during relapse and the improvement after relapse indicate that this test is the most useful metric associated with functional cognitive decrement during relapse.

Analysis of the measures that impacted the cognition improvement after relapse showed that the most important factor was the severity of cognitive impairment during relapse. It seems that the more severe the impairment of any one cognitive domain during relapse, the higher the expected improvement after relapse, especially one month after relapse. Most likely, greater improvement in cognitive scores occurred in patients who had impaired cognition during relapse. Patients who did not experience any cognitive impairment during relapse did not demonstrate an improvement in cognitive scores above their individual norm. These data suggest that actual changes in cognition during relapse and so-called cognitive relapses actually exist. Another important factor affecting cognition was gender. We found a greater improvement of visuospatial memory in men and of verbal memory in women. The differences in the individual cognitive domains between men and women in our study reinforce the possible cognitive differences that were described by other authors: it seems that men may have advantages in visual memory tasks and disadvantages in verbal memory compared to women^29–34^.

In our research, the information processing speed was the only cognitive domain that was not related to gender. However, it was the only measure by which higher scores were associated with higher education and younger age. Our results are in line with other studies that demonstrated that SDMT is the most robust cognitive test that is influenced by demographic variables, such as age, education, and employment^35–37^. The most important clinical factor that affected the improvement of the SDMT score was the EDSS score: the more severe the relapse (higher difference in EDSS score between remission and relapse) and greater the disability before relapse, the lower the improvement of the SDMT score after relapse. In contrast to demographic variables, the relationships between physical and cognitive disability assessed with EDSS and SDMT are not well investigated. Many studies have shown a significant correlation between EDSS and cognitive impairment (not including BICAMS)^38–40^, but it seems that this association reflects a greater cognitive impairment in MS patients with a longer disease duration than disability severity. However, some publications have shown a consistent correlation between SDMT and EDSS. These data show that SDMT has a more robust relationship with physical disability than BVMT-R and CVLT-II^38–41^.

Conversely from SDMT, physical disability had no impact on the BVMT-R or CVLT-II score. However, a greater improvement of visuospatial memory after relapse treatment was found in patients treated with methylprednisolone (not plasma exchange) and in rehabilitated patients, while CVLT-II was more improved in patients with shorter relapse or who received an earlier relapse treatment. The favourable effects of methylprednisolone on the physical disability and MRI parameters, as well as those of an earlier relapse treatment with steroids, are well known. However, the changes in cognition and its different domains after a short methylprednisolone course and in patients with different durations of relapse are not studied enough in MS patients. The better improvement of visuospatial memory after treatment with steroids and the better improvement of verbal memory in patients who received an earlier relapse treatment might indirectly confirm that the impairment of visuospatial and verbal memory can occur during relapse, and, like physical disability, improvement in these cognitive domains is sensitive to treatment with steroids and to earlier treatment. The positive impact of cognitive rehabilitation, contrary to a specific relapse treatment in MS patients, is well known and was described long ago. However, there are limited data on the impact of specific relapse treatments on the impairment of distinct cognitive functions. It is difficult to explain why the visual memory in our study was more sensitive to rehabilitation than the information processing speed and verbal memory. Rehabilitation after MS relapse can improve the BVMT-R and visual memory performance.

## Conclusions

BICAMS was able to detect impairments of the information processing speed during MS relapse. The most significant improvement of cognition was established during the first month after relapse treatment. Various clinical and non-clinical factors impacted the improvement of the BICAMS results after relapse. The main demographic factors that impacted the SDMT score were education and age, while the BVMT-R and CVLT-II scores were influenced by gender. The most important clinical factors were EDSS, which had an impact on the SDMT score; treatment with methylprednisolone, rehabilitation and active interferon-beta, which affected the BVMT-R score; and duration of relapse, which influenced the CVLT-II score. Neuropsychological assessment, like physical disability assessment, is important during relapse. BICAMS may be suitable for quick and effective assessment of cognition during relapse.

## Methods

The study was performed at the Multiple Sclerosis Center of the Vilnius University Hospital Santaros Klinikos. The study protocol was approved by Vilnius Regional Biomedical Research Ethics Committee (Approval No. 158200-13-644-191). All methods were performed in accordance with the relevant guidelines and regulations. Written informed consent was obtained from all participants before inclusion in the study.

### Participants

We enrolled 120 subjects in the prospective follow-up study: 60 relapsing MS patients (MSr), 30 stable MS patients (MSs) and 30 healthy controls (control group, CG). Relapsing MS patients were assessed during relapse and one and three months after relapse. All MS patients were diagnosed with Multiple sclerosis according to the revised McDonald 2010 criteria in usual clinical practice settings by a neurologist of Vilnius Multiple Sclerosis Center. Relapses in MS patients were diagnosed when new neurological symptoms occurred over a minimum of 24 hours and in the absence of a systemic trigger^42^.

All three groups were well matched for age, gender and years of education. Stable MS patients had no MS relapse for at least 3 months before the enrolment. The first symptoms of relapse in relapsing MS patients were separated from the previous relapse by at least 3 months.

The inclusion criteria for all MS patients were:

- Male or female patients older than 18 years of age;
- Diagnosis of Multiple Sclerosis according to the 2010 Revised McDonald criteria;
- Relapsing–remitting course of the disease;
- No assessment with BICAMS in the past;
- No cognition-influencing medication (e.g., antidepressants, neuroleptics, anticholinergic drugs) for at least 1 month prior to the enrolment and during the study;
- Stable and relapsing MS patients had no MS relapse at least 3 months before the assessment;
- All MS patients were steroids and/or plasmapheresis-free for at least 3 months preceding the enrolment.
  The exclusion criteria for MS patients were:
- Any neurologic or psychiatric disorders that could affect cognitive functions;
- History of clinically significant CNS disease (e.g., stroke, traumatic brain or spinal injury) or neurological disorder that could mimic MS;

- Moderate or severe fatigue, anxiety or depression;
- Neurological signs that could interfere with the cognitive performance (e.g., optic neuritis, upper dominant extremity weakness or sever ataxia);
- Unwillingness to cooperate or comply with neuropsychological assessment.

The CG included healthy persons with no history of any cognitive dysfunction, with sight and hearing sufficient for compliance with the study assessment.

### Neurological and comorbidity assessment

Neurological assessment was performed in all MS patients. Neurological disability was assessed with EDSS. MS relapses were recognised in the cases of worsening or new neurological symptoms appearance that lasted for at least 24 hours^42^. Fatigue was assessed with the Fatigue Severity Scale^43^, depression with the Hospital Anxiety and Depression Scale^44^. Cut-off points of 4/9 and 12/21 were identified as indicative of possible fatigue and depression, respectively, and these patients were excluded. To estimate the severity of the relapse, the EDSS score in relapsing MS patients was collected from the medical records during the last visit to the centre that was performed not more than 3 months before the baseline assessment. It was possible to assess the EDSS score retrospectively in 49 relapsing MS patients. The duration of the relapse was considered as the period from the first neurological symptom appearance until the relapse treatment.

### Neuropsychological assessment with BICAMS

All subjects were assessed by the same person and in the same assessment room. Relapsing MS patients were examined during the relapse and one and three months after relapse. The visits were performed within the allowed visit window of ±3 days. Assessment during relapse was performed before the relapse treatment. The relapses were treated by the treating physician according to the standard clinical practice and clinical situation: steroids and/or plasmapheresis was given for the treatment. The cognition-evaluating physician had no influence on the relapse treatment options.

All subject were examined with BICAMS in the same sequence^10,11^:

1. The Symbol Digit Modalities Test (SDMT);
2. The Brief Visuospatial Memory Test Revised (BVMT-R), first 3 recall trials;
3. The California Verbal Learning Test, second edition (CVLT-II), first 5 trials. The Lithuanian version of CVLT-II was used for the assessment.

**Figure 1:**
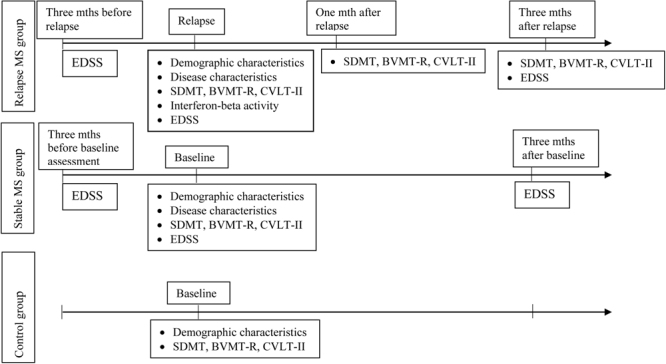
Study design and timeline of the assessments. MS - multiple sclerosis, mth(s) – months, EDSS – Expanded Disability Status Scale, SDMT – Symbol Digit Modalities Test, BVMT-R - Brief Visuospatial Memory Test-Revised, CVLT-II – California verbal learning test, II ed.

The different versions of BVMT-R were used during relapse and at one and three months. The first available and recommended version of CVLT-II was used during relapse, the second one at one month after relapse, and the first one was repeated at three months (Fig. 1). Four relapsing MS patients were not included in the final data analysis due to the inability to come to the scheduled visits.

### Immunomodulatory treatment and immunological assessment of interferon-beta activity

The patients received and continued their immunomodulatory treatment according to the clinical situation and to the treatment guidelines established by the Lithuanian Ministry of Health (order No. V-1129). In patients who were treated with interferon-beta for at least 1.5 years, the biological activity of interferon-beta was assessed by measuring the marker of interferon-beta activity, myxovirus resistance protein A (MxA). MxA mRNA expression <0.586 before interferon-beta injection and <3.84 after INF-beta injection were considered negative. Biological responders, poor biological responders, and biological non-responders were defined based on the absolute values of their MxA expression/induction indicators regarding those 2 cut-off values, which were established before the study. Biological responders were defined as the patients whose MxA expression values were above both threshold levels. In case only 1 threshold was reached, a patient was assigned to the group of poor biological responders. If neither of the 2 thresholds was reached, a patient was defined as a biological non-responder^45–47^.

### Methods of statistical analysis

Data were analysed using the statistical software package SPSS (version 20.0 for Windows). Descriptive statistics are presented as the mean (m) and standard deviation (±SD). Student’s t-test was used to compare means of the same variables in two groups when their distribution was normal. Categorical variables were analysed using the chi-square or Fisher’s exact test. To check the normality of distribution of quantitative variables, the Shapiro-Wilk test was used.

One-way analysis of variance (ANOVA) was used to compare the means of relapsing MS patients, stable MS patients and CG. To determine the significance of pairwise comparisons in one-way ANOVA, Bonferroni’s post hoc test (for equal variances, Levene’s test p > 0.05) or Tamhane’s test (for unequal group variances, Levene’s test p < 0.05) was used.

General linear model (GLM) repeated measures were used when assessing the data of relapsing MS patients at different time points (relapse and one and three months after relapse). Linear regression was used to assess the impact of different demographic, clinical and immunological factors on cognition.

Significance level was accepted at p < 0.05.

## References

[1] Rao SM, Leo GJ, Bernardin L, Unverzagt F (1991). Cognitive dysfunction in multiple sclerosis. I. Frequency, patterns, and prediction. Neurology..

[2] Amato MP, Ponziani G, Siracusa G, Sorbi S (2001). Cognitive dysfunction in early-onset multiple sclerosis: a reappraisal after 10 years. Arch Neurol..

[3] Langdon DW (2011). Cognition in multiple sclerosis. Curr Opin Neurol..

[4] Julian LJ, Vella L, Vollmer T, Hadjimichael O, Mohr DC (2008). Employment in multiple sclerosis. Exiting and re-entering the work force. Journal of Neurology..

[5] Larocca N, Kalb R, Scheinberg L, Kendall P (1985). Factors associated with unemployment of patients with multiple sclerosis. Journal of Chronic Diseases..

[6] Moccia M (2016). Cognitive impairment at diagnosis predicts 10-year multiple sclerosis progression. Mult Scler..

[7] Kizlaitiene R, Kaubrys G, Giedraitiene N, Ramanauskas N, Dementaviciene J (2017). Composite Marker of Cognitive Dysfunction and Brain Atrophy is Highly Accurate in Discriminating Between Relapsing-Remitting and Secondary Progressive Multiple Sclerosis. Med Sci Monit..

[8] Rao, S. M. The Cognitive Function Study Group of the National Multiple Sclerosis Society A. Manual for the Brief Repeatable Battery of Neuropsychological Tests in Multiple Sclerosis. (Milwaukee, 1990).

[9] Benedict RH (2002). Minimal neuropsychological assessment of MS patients: a consensus approach. The Clinical Neuropsychologist..

[10] Langdon DW (2012). Recommendations for a Brief International Cognitive Assessment for Multiple Sclerosis (BICAMS). Mult Scler..

[11] Benedict RHB (2012). Brief International Cognitive Assessment for MS (BICAMS): international standards for validation. BMC Neurol..

[12] Niccolai C (2015). A comparison of the brief international cognitive assessment for multiple sclerosis and the brief repeatable battery in multiple sclerosis patients. BMC Neurol..

[13] Kurtzke JF (1983). Rating neurologic impairment in multiple sclerosis: An expanded disability status scale (EDSS). Ann Neurol..

[14] Hobart J, Freeman J, Thompson A (2000). Kurtzke scales revisited: the application of psychometric methods to clinical intuition. Brain..

[15] Marrie RA, Goldman M (2007). Validity of performance scales for disability assessment in multiple sclerosis. Mult Scler..

[16] Benedict RHB (2014). Characterizing cognitive function during relapse in multiple sclerosis. Mult Scler..

[17] Prado FM, Kosac VA, Dib JG (2012). Cognitive relapse in multiple sclerosis: Report of 3 cases. Mult Scler..

[18] Pardini M (2014). Isolated cognitive relapses in multiple sclerosis. J Neurol Neurosurg Psychiatry..

[19] Morrow SA, Jurgensen S, Forrestal F, Munchauer FE, Benedict RH (2011). Effects of acute relapses on neuropsychological status in multiple sclerosis patients. J Neurol..

[20] Dusankova JB, Kalincik T, Havrdova E, Benedict RH (2012). Cross cultural validation of the Minimal Assessment of Cognitive Function in Multiple Sclerosis (MACFIMS) and the Brief International Cognitive Assessment for Multiple Sclerosis (BICAMS). Clin Neuropsychol..

[21] Goretti B (2014). The Brief International Cognitive Assessment for Multiple Sclerosis (BICAMS): normative values with gender, age and education corrections in the Italian population. BMC Neurol..

[22] Sandi D (2015). The Hungarian validation of the Brief International Cognitive Assessment for Multiple Sclerosis (BICAMS) battery and the correlation of cognitive impairment with fatigue and quality of life. Mult Scler Relat Disord..

[23] Giedraitiene N, Kizlaitiene R, Kaubrys G (2015). The BICAMS Battery for Assessment of Lithuanian-Speaking Multiple Sclerosis Patients: Relationship with Age, Education, Disease Disability, and Duration. Med Sci Monit..

[24] O’Connell K, Langdon D, Tubridy N, Hutchinson M, McGuigan C (2015). A preliminary validation of the brief international cognitive assessment for multiple sclerosis (BICAMS) tool in an Irish population with multiple sclerosis (MS). Mult Scler Relat Disord..

[25] Spedo CT (2015). Cross-cultural Adaptation, Reliability, and Validity of the BICAMS in Brazil. Clin Neuropsychol..

[26] Erlanger DM (2014). Reliability of a cognitive endpoint for use in a multiple sclerosis pharmaceutical trial. J Neurol Sci..

[27] Rao, S. M. *et al*. Correlations between MRI and information processing speed in MS: A meta-analysis. *Mult Scler Int*. Article 975803 (2014). 10.1155/2014/975803 PMC3984845 24795824

[28] Benedict RH (2017). Validity of the Symbol Digit Modalities Test as a cognition performance outcome measure for multiple sclerosis. Mult Scler..

[29] Li R (2014). Why women see differently from the way men see? A review of sex differences in cognition and sports. J Sport Health Sci..

[30] Pauls F, Petermann F, Lepach AC (2013). Gender differences in episodic memory and visual working memory including the effects of age. Memory..

[31] Proust-Lima C (2008). Gender and education impact on brain aging: a general cognitive factor approach. Psychol Aging..

[32] Heinzel S (2013). Aging-related cortical reorganization of verbal fluency processing: a functional near-infrared spectroscopy study. Neurobiol Aging..

[33] Murre JM, Janssen SM, Rouw R, Meeter M (2013). The rise and fall of immediate and delayed memory for verbal and visuospatial information from late childhood to late adulthood. Acta Psychol (Amst)..

[34] Munro CA (2012). Sex differences in cognition in healthy elderly individuals. Neuropsychol Dev Cogn B Aging Neuropsychol Cogn..

[35] Smith, A. Symbol Digits Modalities Test. *Western Psychological Services*. (Los Angeles, 1982).

[36] Uchiyama CL (1995). Longitudinal comparison of alternate versions of the Symbol Digit Modalities Test: Issues of form comparability and moderating demographic variables. The Clinical Neuropsychologist..

[37] Yeudall LT, Fromm D, Reddon JR, Stefanyk WO (1986). Normative data stratified by age and sex for 12 neuropsychological tests. Journal of Clinical Psychology..

[38] Sepulcre J (2006). Cognitive impairment in patients with multiple sclerosis using the Brief Repeatable Battery-Neuropsychology test. Mult Scler..

[39] Duque B (2008). Memory decline evolves independly of disease activity in MS. Mult Scler..

[40] Deloire M, Ruet A, Hamel D, Bonnet M, Brochet B (2010). Early cognitive impairment in multiple sclerosis predicts disability outcome several years later. Mult Scler..

[41] Vanotti S (2015). Normatization of the symbol digit modalities test: oral version in a Latin American country. Appl Neuropsychol Adult..

[42] Schumacker GA (1965). Problems of experimental trials of therapy in multiple sclerosis: Report by the panel on the evaluation of experimental trials of therapy in multiple sclerosis. Ann N Y Acad Sci..

[43] Krupp LB (1989). The fatigue severity scale. Application to patients with multiple sclerosis and systemic lupus erythematosus. Arch Neurol..

[44] Zigmond AS, Snaith RP (1983). The hospital anxiety and depression scale. Acta Psychiatr Scand..

[45] Pachner AR, Narayan K, Pak E (2006). Multiplex analysis of expression of three IFNbeta-induced genes in antibody-positive MS patients. Neurology..

[46] Gilli F (2005). Biological responsiveness to first injections of interferon-beta in patients with multiple sclerosis. J Neuroimmunol..

[47] Giedraitiene N (2015). Therapeutic Plasma Exchange in Multiple Sclerosis Patients with Abolished Interferon-beta Bioavailability. Med Sci Monit..

